# Hemifacial microsomia: a scoping review on progressive facial asymmetry due to mandibular deformity

**DOI:** 10.1007/s10006-024-01276-5

**Published:** 2024-07-02

**Authors:** Peterson Makinde Atiba, Bukola Rukayat Omotoso, Anil Madaree, Lelika Lazarus

**Affiliations:** 1 Discipline of Clinical Anatomy, College of Health Sciences, University of KwaZulu-Natal, Westville Campus, Durban, South Africa; 2 Department of Plastic and Reconstructive Surgery, Inkosi Albert Luthuli Central Hospital, Durban, South Africa; 3 Anatomy Programme, Faculty of Basic Medical and Health Sciences, College of Health Sciences, Bowen University, Iwo, Osun State Nigeria

**Keywords:** Hemifacial microsomia, Progressive facial asymmetry, Mandible, Mandibular morphometrics

## Abstract

**Purpose:**

This scoping review explores various parameters of the mandible in progressive facial asymmetry (FA) in hemifacial microsomia (HFM) patients, highlighting its relationship with sex, population, and age group.

**Methods:**

The review was based on a comprehensive search of PubMed, EBSCOhost, and Web of Science. Eligible studies that met the inclusion criteria form part of the selection study. The included studies were appraised using screening and quantitative criteria of mixed-method appraisal tools. The authors utilised a pre-set data extraction form to obtain information from the included studies.

**Results:**

Eleven studies met the inclusion criteria. The mandible parameters used were angular measurements, chin point, ramal height, body length, and total length. There was no relationship between FA and sex in HFM patients in the included studies. Most of the studies were comprised of European participants (55%), followed by Americans (36%) and Chinese (9%). The age groups included in the selected studies were categorised as dentition age (18%), early-to-middle childhood (18%), and varied ages (64%). The data presented in this review only pertains to the anomalous characteristics recorded on the affected side in HFM patients. No concomitant control data was recorded in this review.

**Conclusion:**

An assessment of the included studies revealed that FA does not increase with age in HFM. Hence, FA is non-progressive in HFM patients. This information is relevant to diagnosing and managing HFM patients. More reports are needed on the progression of FA in HFM patients.

**Supplementary Information:**

The online version contains supplementary material available at 10.1007/s10006-024-01276-5.

## Introduction

Hemifacial microsomia (HFM) is a congenital anomaly in which one-half of the face does not develop normally. HFM is derived from the first and second pharyngeal arches malformation [1]. It is the second most prevalent congenital craniofacial defect after cleft lip and palate (1: 3,500-5,600 in the United States of America) [2]. It has been speculated to be due to genetic, maternal, and environmental conditions leading to stapedial artery injury (replaced by an external carotid system in adults), dysgenesis of Meckel’s cartilage and aberrant migration of neural crest cells [3]. This defect is more evident in the jaw and ear areas, though the involvement of the eye, cheek, nerve, soft tissue, other cranial parts, and neck may accompany it. HFM can also be described as skeletal or soft-tissue defects or a combination of skeletal and soft-tissue defects [4]. Although HFM implies facial involvement only, individuals with HFM often have associated extracranial defects such as neurological, cardiac, genitourinary, pulmonary, gastrointestinal, and skeletal malformations [5–9]. In a clinical presentation, mandibular hypoplasia has been the cornerstone of this deformity with unilateral or bilateral microtia. Craniofacial growth in the affected patients depends on the extent of the deformity [1]. Mandibular size, shape, and location to the maxilla and base of the skull are determined by bone deposition and resorption on the periosteal and endosteal surfaces [10, 11]. The increase in vertical height of the ramus and its posterior movement occurs due to bone deposition on the posteroinferior surface and resorption on the anterior surface [10]. Resorption occurring along the anterior border of the ramus also adds to the length of the mandibular body [10, 11]. The mandibular body, the arch’s shape and width are determined by resorption on the medial surface and deposition on the lateral surface [10]. The mandibular skeletal midline deviates to the affected side with unilateral growth impairment [11].

During the 4th week of embryonic life, distinct face development is identifiable from five facial primordia structures surrounding the stomodeum [12–14]; these include one frontonasal prominence and paired maxillary and mandibular processes [15, 16]. The latter two processes originate from the first branchial arch [16, 17]. The innervation to the face arises from the first and second pharyngeal arches, while the blood vessels are derived from the third aortic arch [17]. All these develop to form the structures of the future face [16, 17]. Disruption in the migration and differentiation pathway of the pluripotent neural crest cells results in congenital abnormalities [12, 14].

Facial asymmetry (FA) classification includes congenital, acquired, or developed from an unknown aetiology [18]. HFM is one of the congenital disabilities responsible for facial asymmetry. Asymmetry of the mandibles could involve the ramus, the condyle, the body, and symphysis, which may result in size, volume, or position changes. Skeletal deviation equal to or greater than 4 mm is considered asymmetry noticeable in an individual’s face, while a skeletal deviation less than 4 mm is identified as mild and unnoticeable [19, 20]. In addition to skeletal deformity, soft tissue thickness also influences facial disproportion; hence, a deviation equal to or greater than 2 mm of the soft tissue is marked as facial asymmetry [21, 22].

Some authors hypothesised that mandibular growth defect contributes to FA in HFM patients [23]. Other authors have suggested that mandibular growth defect does not influence FA in HFM patients. The indicators of FA include changes in the maxillary occlusal plane, piriform rim angle, intergonial angle, and chin point deviation [24, 25]. Hence, there are contrasting data on the influence of mandibular growth defects on FA in HFM.

Previous reviews focused on FA, temporomandibular disorders, and mandibular asymmetry. However, only a few discussed the progression of FA due to mandibular disproportion (asymmetry) in HFM patients [23, 26–29]. The knowledge of skeletal and soft tissue defects is fundamental in the reconstructive approach to facial surgery [30, 31]. This review aims to map out various parameters of the mandible (i.e., the deviation of occlusal plane, piriform angle, intergonial angle, chin point, mandibular ramus height, body length and total mandibular length) in the progression of FA in HFM patients, highlighting its relationship with sex, population, and age group.

## Materials and methods

A scoping review based on the framework developed by Arksey and O’Malley [32] and further expanded by Levac et al. [33] was used in this analysis. The literature on the progression of FA due to the mandibular morphology and disproportion (asymmetry) in HFM patients was extensively searched. The following steps were taken in the search process: identifying the research question, identifying relevant studies, study selection, charting the data, and collating, summarising, and reporting the results.

### Research question

What were the available studies on the progression of FA due to a deformed mandible in HFM patients?

#### Sub–question

- In which countries were these studies completed?
- What age range or grouping was used to report these studies?
- To determine facial asymmetry, what parameters were used (i.e., changes in the maxillary occlusal plane, piriform rim angle, intergonial angle, chin point deviation, mandibular ramal height, mandibular body length, total mandibular length)?

### Search strategies

A systematic search of the literature was conducted on PubMed (National Centre of Biotechnology Information, Bethesda, Maryland, United States), EBSCOhost (EBSCO Information Services, *Ipswich*, Massachusetts, USA), and Web of Science (Clarivate Analysis, PLC, Philadelphia, Pennsylvania, USA). The keywords used for this search include hemifacial microsomia, progressive facial asymmetry, mandibular asymmetry, mandibular growth restriction, mandibular hypoplasia, facial asymmetry, and mandibular morphology. These keywords were used in combination with the Boolean term (AND, OR), such as “hemifacial microsomia AND facial asymmetry” OR “hemifacial microsomia AND mandibular growth restriction” OR “hemifacial microsomia AND mandibular growth” OR “hemifacial microsomia AND mandibular hypoplasia” OR “hemifacial microsomia AND mandibular asymmetry” OR “hemifacial microsomia AND mandibular morphology” OR “facial asymmetry AND mandibular morphology” OR “facial asymmetry AND mandibular hypoplasia” OR “hemifacial microsomia AND progressive facial asymmetry” OR “progressive facial asymmetry AND mandibular asymmetry”. An initial limited search of PubMed was completed by analysing the text words in titles, abstracts, and index terms used to describe articles (see Appendix I). A second search using identified keywords and index terms was used across all databases. The third step search was also conducted across all databases using a reference list of all identified articles. The most recent search of this review was accessed on 31st December 2023.

The scoping review results were reported using the PRISMA (Preferred Reporting Items for Systematic reviews and Meta-analysis) guidelines for Scoping review.

#### Inclusion criteria

The inclusion criteria included studies on the progression of FA in HFM. Original articles on mandibular asymmetry, morphology, and hypoplasia between 1969 and 2022. Articles, reports, and books available in English, articles already translated into English, and articles in dual languages formed part of this review.

#### Exclusion criteria

Research articles, reviews, and reports on the progression of FA in HFM before 1969 were excluded during study selection (Pruzansky reported the first study on mandibular hypoplasia classification in 1969). Reviews of HFM in progressive FA were excluded. Studies not primarily on the progression of FA in HFM and animal-based HFM studies did not form part of this review.

### Study selection

A set of questions aligned with the study’s objective assessed the relevant studies identified during the literature search. Following the search, all identified citations were collated and uploaded into EndNote 20 (Clarivate Analytics, PA, USA), and duplicates were removed. Study selection was made by two authors (PMA, BRO), who screened titles and abstracts of all retrieved studies to assess eligibility. When eligibility could not be determined, full articles were retrieved.

### Quality appraisal

The quality appraisal of the included studies was conducted using screening criteria for all studies (Table 1). Quantitative descriptive criteria of the Mixed Methods Appraisal Tool (MMAT)-Version 2018 were used for the included studies [34]. The appraisal was done by colleagues (OA and OF), not part of this review. A score of 20% is given when an eligible study fulfils one quantitative criterion, 40% if it fulfils two criteria, 60% if it fulfils three criteria, 80% if it fulfils four criteria, and 100% if it fulfils all quantitative criteria.

**Table 1:** Mixed Methods Appraisal Tool (MMAT), version 2018 indicators for screening questions and quantitative descriptive studies (adapted from Hong et al. [34])

Category of study designs	Methodological quality criteria
Screening questions	*1*. *Are there clear research questions or objectives*?
	*2*. *Do the collected data allow to address the research questions*?
Quantitative descriptive studies	*1*. *The sampling strategy is relevant to address the research questions*
	*2*. *The sample is a representative of the population study*
	*3*. *Appropriate and validated measurements*
	*4*. *Low risk of nonresponse bias*
	*5*. *Appropriate statistical analysis was done to answer the research question*

Note: Studies were deemed acceptable when the first two screening questions for all study types and at least one of the indicators in the category of the study designs were met

### Data extraction and analysis

A data extraction form was used to extract details on characteristics of the included studies by two authors (PMA, BRO), such as author name, date of publication, population, sample size, male (%), female (%), age range, aim of the study, methodology, and significant findings. The extraction form was subjected to review. Co-authors LL and AM independently used this form to extract data from all eligible studies. Microsoft Excel 2019 was used to compile all data on a spreadsheet. The content analysis of each article included in the review was done.

## Results

### Description of included studies

**Fig. 1:**
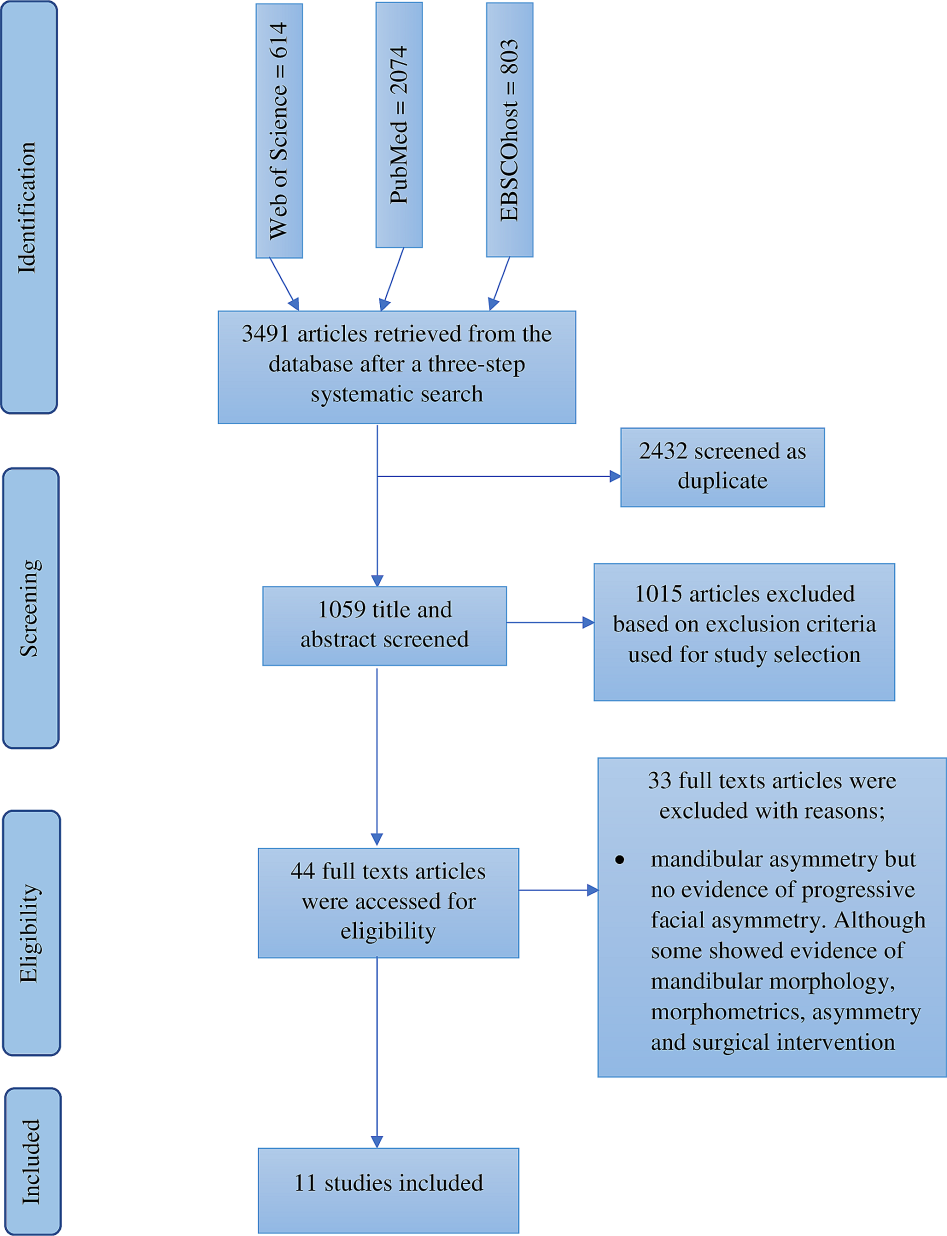
Flow diagram of study selection

A total of 3491 articles were identified during the literature search, including research papers, reports, and books. Two thousand four hundred and thirty-two duplicates were removed. After screening titles and abstracts, 1015 articles were excluded based on the exclusion criteria. Forty-four full-text articles that met the eligibility criteria were reviewed. Thirty-three articles were excluded because they lacked evidence on the progression of FA, although they showed evidence of mandibular morphology, morphometrics, asymmetry, and surgical intervention. However, eleven articles were eligible and included in this review (Fig. 1).

**Fig. 2:**
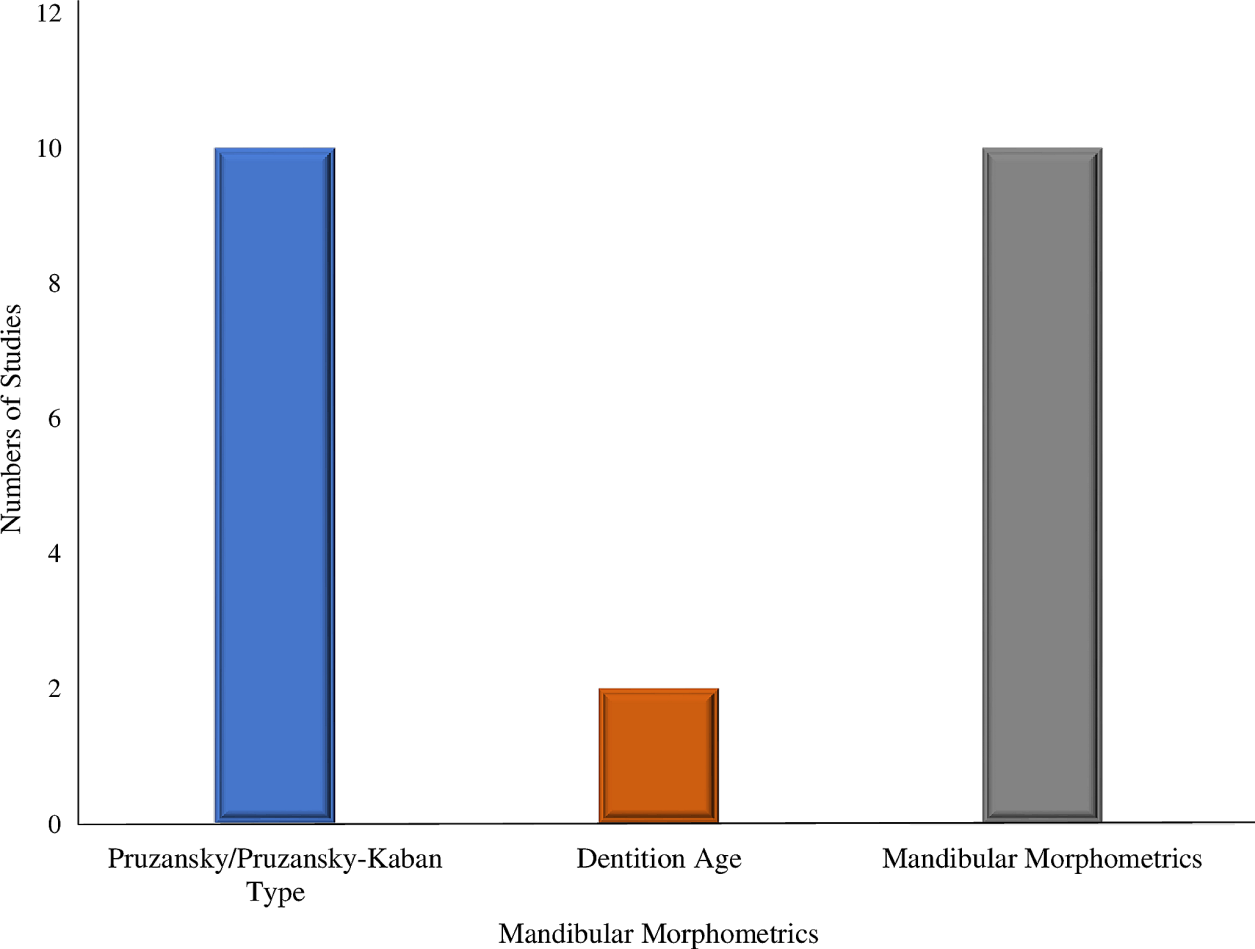
Number of studies suggesting the progression of facial asymmetry in relation to Pruzansky-Kaban type classification, dentition age range and mandibular morphometrics in the literature from 1969 to date. The X-axis shows the classification, dentition age and measurement of the deformed mandible, and the Y-axis illustrates the number of studies

**Fig. 3:**
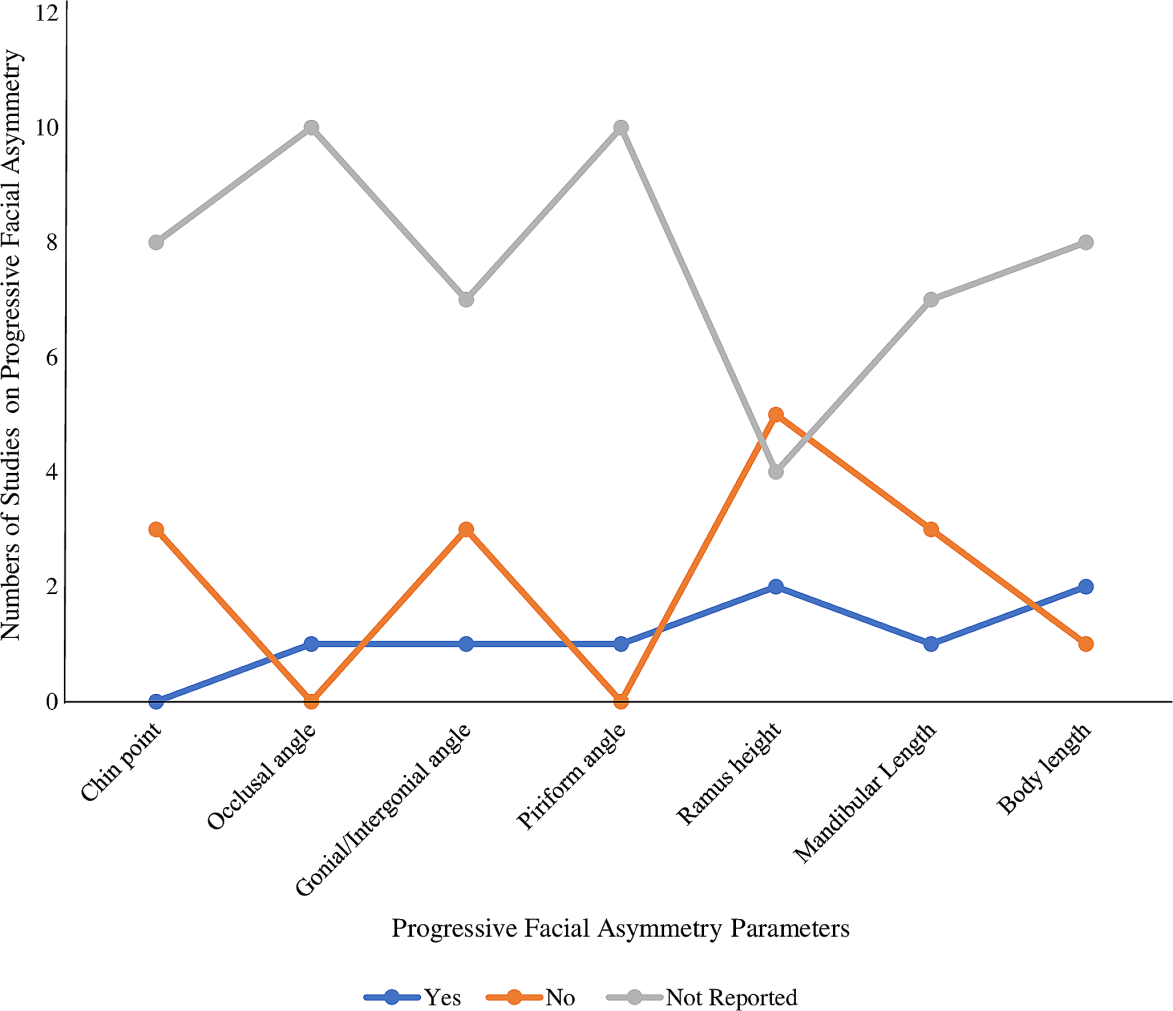
Line graph showing assessment of all the included studies based on the facial asymmetry parameters in hemifacial microsomia patients. The X-axis represents facial asymmetry parameters, and the Y-axis represents the number of studies showing the progression of facial asymmetry

Table 2 summarises the included studies and their significant findings. Table 3 shows the frequency distribution of FA parameters in the included studies. Figure 2 shows the level of knowledge of included studies on the progression of FA in relation to Pruzansky-Kaban type classification, dentition age range and mandibular morphometrics. Figure 3 illustrates an assessment of all the included studies based on the facial asymmetry parameters in hemifacial microsomia patients. Most (55%) of the included studies were conducted in Europe [25, 35–39], 36% were conducted in the United States of America [24, 40–42], and 9% were conducted in the People’s Republic of China [43]. The largest sample size is 210 [43], while the lowest is 7 [40].

**Table 2:** Characteristics of included studies

Author (Year)	Population	Sample size	Male %	Female %	Age Range	Aim of Study	Methodology (Cross-sectional/Longitudinal study)	Major Finding	Progressive Facial asymmetry (Yes/No)	MMAT Score
**Rune*et al***., **1981**	Sweden	11	64	36	3–14 years	The displacement of the mandible and the maxillary bones with growth was studied in 11 children with HFM.	Roentgen stereophotogrammetry with metallic implants was utilised to record growth relative to the frontal bone. (Longitudinal study)	No correlation was found between the extent of the mandibular deformity, as seen on orthopantomograms, and the displacement of the mandible with growth.	No	80%
**Polley** ***et al***., 1997*	USA	26	58	42	3.1–16.7 years (mean age)	To analyse mandibular skeletal growth longitudinally in unoperated hemifacial microsomia patients from childhood to adolescence and determine whether asymmetry improves, remains constant or progresses during development.	Posteroanterior cephalograms were used to evaluate each patient’s horizontal and vertical mandibular asymmetry. (Longitudinal study)	The skeletal mandibular asymmetry in hemifacial microsomia is not progressive, and the growth of the affected side in these patients parallels that of the non-affected side. The grade and the side of the mandibular deformity did not progress with time.	No	80%
**Kusnoto** ***et al***. 1999*	USA	7	66	33	14–19 years	To analyse the longitudinal growth patterns of unoperated patients before and after distraction osteogenesis in three dimensions.	Lateral and posteroanterior cephalograms were used preoperatively for nine years. Computerised three-dimensional models were constructed from lateral and posteroanterior using a vector intercept algorithm. (Longitudinal study)	Unoperated patients with HFM maintain asymmetric facial proportions and do not worsen with time. There was a reversal to the initial presentation 18 months after distraction osteogenesis.	No	60%
**Kearns** ***et al***., 2000*	USA	67	-	-	Deciduous dentition: 3.4–4.1 years (mean age) Mixed dentition: 8.0-8.6 (mean age) Permanent dentition: 21 (mean age)	To test the hypothesis that facial asymmetry in HFM is a progressive deformity.	A retrospective review of medical records and posteroanterior cephalometrics radiographs in HFM patients. Mandibular asymmetry was determined by measurement angles between the actual horizontal plane and the following planes: piriform rim, maxillary occlusal plane, and intergonial angle. (Cross-sectional study)	The data from the study suggested that facial asymmetry is progressive by increasing the piriform rim, maxillary occlusal plane, and intergonial angles. Surgical correction is advocated during the mixed dentition stage.	Yes	80%
**Meazzini** ***et al***., 2012*^#^	Italy	22	-	-	5–7 years	To compare children with HFM, a group was treated with distraction osteogenesis (either during deciduous or mixed dentition), and another group was not treated until the completion of growth.	Mandibular vertical changes were measured on panoramic radiographs taken at different points in time. (Longitudinal study)	Untreated hemifacial microsomia patients maintained facial proportions throughout development. Esthetics and psychological gains were observed but lost due to inherent genetic factors. There was a relapse of facial asymmetry at 12 months of treatment.	No	80%
**Ongkosuwito** ***et al***., 2013a*	Netherlands	84	44	56	9.9 years (mean age)	To design mandibular ramal height growth curves for patients with HFM and compare them with Dutch controls.	To determine the mandibular ramal distances using an orthopantomogram of 84 HFM patients and compare with 329 healthy individuals without hemifacial microsomia. (Longitudinal study)	HFM patients start and end with smaller mandible but grow like the healthy population. Therefore, growth is similar in both HFM patients and healthy populations.	No	80%
**Ongkosuwito** ***et al***., 2013b*	Netherlands	75	48	52	4–29 years	To design a craniofacial linear growth curve for an unoperated mandible with HFM and Dutch controls.	A cephalometrics analysis of hemifacial microsomia in a longitudinal study on serial lateral cephalograms. (Longitudinal study)	Patients with HFM have more vertical and retruded growth than control as severity increases. The growth curve is estimated for each patient to determine the best treatment age from a growth perspective. Hence, an individual approach is advocated.	No	80%
**Renkema** ***et al***., 2022*	Netherlands	110	51	49	3–56 years	To investigate the potential progressiveness of mandibular asymmetry and to study factors that influence chin point deviation in patients with unilateral craniofacial microsomia (CFM).	Radiographic images and medical photographs of patients with unilateral craniofacial microsomia were utilised. Clinical photographs were used to determine chin point deviation. (Longitudinal study)	A linear mixed model for repeated measurements showed no significant association between age and chin point deviation. Although an association exist in the Pruzansky-Kaban score between sex and chin point deviation. The potential progressiveness of CFM could not be affirmed.	No	80%
**Kapiro** ***et al***., 2023*	Finland	40	-	-	Mixed dentition: 7.4 ± 1.49 years (mean age) Late mixed dentition: 10.9 ± 1.63 (mean age) Permanent dentition: 17 ± 5.23 (mean age)	To describe craniofacial microsomia (CFM) patients’ mandibular growth from early childhood to adolescence with attention to symmetry.	The assessment of ramus height in anteroposterior and panoramic radiographs, mandible length in anteroposterior radiographs, maxillary protrusion, and mandibular retrognathia in lateral cephalograms were conducted across four distinct age cohorts. (Cross-sectional study)	Findings suggest that mild-type CFM is not progressive. During growth, mandibular asymmetry measured in the horizontal, vertical and sagittal planes did not increase.	No	80%
**Shetye** ***et al***., 2023*	USA	30	70	30	5-14years	To examine the growth rate discrepancy of the affected and unaffected ramus heights in Pruzansky Type I and Type II mandibles.	A serial retrospective longitudinal growth study of untreated patients with HFM (Unilateral Craniofacial) classified based on Pruzansky type I and II mandible, with a mean follow-up of 3.7 years. (Longitudinal study)	The growth rate discrepancy of the affected and unaffected ramus heights was more severe in Pruzansky Type II mandibles than in Pruzansky Type I mandibles. This study suggests the progressive nature of facial asymmetry in Pruzansky Type II mandibles.	Yes	80%
**Zhang** ***et al***., 2023*	China	210	60	40	0.2–11 years	To quantitively analyse the mandibular ramus and body deformities, assessing the asymmetry and progression in different components.	Linear and volumetric measurements of the ramus and the body were collected via their preoperative imaging data (using three-dimensional computed tomography) to compare the different sides and severities. (Cross-sectional study)	There were asymmetries in the mandibular ramus and body regions, which involved the ramus more. A significant contribution to progressive asymmetry from the body suggests treatment focus in this region.	Yes	80%

*Studies that reported the severity of HFM according to Pruzansky or Pruzansky-Kaban classification

^#^Studies reported on follow-up period (range between eighteen months to ten years and above)

All the included studies assessed FA changes in the overall HFM sample

**Table 3:** The frequency table shows the parameters of facial asymmetry in the eleven included studies

Parameters	Numbers of articles (%)
Chin Point	27 ^25, 39, 40^
Ramus Height	64 ^35, 36, 39–43^
Total Mandibular Length	27 ^40, 41, 43^
Mandibular body length	27 ^40–42^
Occlusal plane angle	27 ^24, 37, 38^
Piriform rim angle	9 ^24^
Gonial/Intergonial angle	27 ^24, 39, 40, 43^

**Table 4:** Mandibular parameters used to measure facial asymmetry and treatment in selected studies

Author(s)	Mandibular parameters to measure FA	Received HFM treatment	Treatment modality	Age at treatment (years)	Treatment duration (years)	Treatment outcome on FA
Rune et al. 1981	occlusal plane angle	No	Nil	Nil	Nil	Nil
Polley et al. 1997	chin point, ramus height, total mandibular length, mandibular body length, gonial/intergonial angle	No	Nil	Nil	Nil	Nil
Kusnoto et al. 1999	chin point, ramus height, gonial/intergonial angle	Yes	distraction osteogenesis	5.9 ± 0.8	5	The initial gain was a loss due to the relapse facial asymmetry at the completion of growth
Kearns et al. 2000	occlusal plane angle, piriform rim angle, gonial/intergonial angle	No	Nil	Nil	Nil	Nil
Meazzini et al. 2012	ramus height	Yes	distraction osteogenesis	12.5 ± 2.4	1.5	Improve facial symmetry, but the affected and unaffected grew at the same rate before treatment
Ongkosuwito et al. 2013a	ramus height	No	Nil	Nil	Nil	Nil
Ongkosuwito et al. 2013b	occlusal plane angle	No	Nil	Nil	Nil	Nil
Renkema et al. 2022	chin point	No	Nil	Nil	Nil	Nil
Kapiro et al. 2023	ramus height, total mandibular length, gonial/intergonial angle	No	Nil	Nil	Nil	Nil
Shetye et al. 2023	ramus height, total mandibular length, mandibular body length	No	Nil	Nil	Nil	Nil
Zhang et al. 2023	ramus height, mandibular body length	No	Nil	Nil	Nil	Nil

All included studies were observational; 82% were retrospective studies [24, 25, 36, 37], while 18% were prospective studies [35, 38]. Only three of the included studies (27%) were cross-sectional [24, 39, 43], while the remaining (82%) were longitudinal studies [25, 35–42] (Table 2). A total of 67% of the included studies described sex in their report [25, 36–41, 43]. Age grouping varied among the included studies; two (18%) utilised dentition age grouping [24, 39], two (18%) utilised infancy to childhood [41, 43], while others (64%) used varied age grouping [25, 35–40, 42]. Three (27%) of the included studies suggested the progression of facial asymmetry [24, 42, 43], while the remaining (73%) suggested that FA remains constant in HFM patients [25, 35–41].

### The methodological quality of the included studies

The eleven included studies were deemed good quality as they answered the first two screening questions and fulfilled at least three quantitative criteria of MMAT. Regarding the quantitative criteria, one study met three criteria- 60% and ten met four criteria- 80% (Table 2).

Table 3 shows the frequency distribution of FA parameters in the eleven included studies. The more common parameters used in the included studies were: 64% used ramus height [35, 36]. 27% used chin point [25, 40, 41], 27% used occlusal plane angle [24, 37, 38], 36% used gonial/intergonial angle [24, 39–41], 27% used total mandibular length [39–41], 27% used mandibular body length [41–43]. In addition, a few of the included studies discussed the treatment approach and outcome (Table 4).

### Studies suggesting evidence of progressive facial asymmetry in relation to Pruzansky-Kaban type classification, dentition age range and mandibular morphometrics

Ten (91%) of the included studies utilised either Pruzansky [35, 39–42] or Pruzansky-Kaban [24, 25, 36, 37, 43] types to classify the mandibular asymmetry in HFM. Two (18%) of the included studies utilised dentition age [24, 39], two (18%) of the included studies utilised infancy to childhood stage [41, 43] for age grouping. The majority (91%) of the included studies showed evidence of knowledge of mandibular morphometry in HFM patients [24, 25, 35–37].

### Assessment of all the included studies based on their conclusion on the progressiveness of FA in hemifacial microsomia patients

An assessment of mandibular morphometric parameters was used to determine the progressiveness of FA in the HFM population in all (100%) of the included studies [24, 25, 35–39]. An increase in the values of the parameters is associated with progressive FA and was denoted as ‘Yes’ (Fig. 3). These include occlusal plane/angle [24], gonial/intergonial angle [24], piriform angle [24], ramus height [42, 43], mandibular length [42], and body length [42, 43].

A decrease or no change in the parameters’ values is associated with FA’s non-progressiveness and was denoted as ‘No’ (Fig. 3). These include chin point [25, 38, 41], gonial/intergonial angle [39–41], ramus height [35, 36, 39–41], mandibular length [38, 39, 41] and body length [40].

Some studies did not report on these parameters (Fig. 3). These include chin point [24, 35–37, 39, 40, 42, 43], occlusal plane/angle [25, 35–39], gonial/intergonial angle [25, 35–38, 42, 43], piriform angle [25, 35–39], ramus height [24, 25, 37, 38], mandibular length [24, 25, 35–37, 40] and body length [24, 25, 35–39, 41, 43].

## Discussion

This review seeks to map out the various parameters of the mandible in the progression of FA in HFM patients, highlighting its relationship with sex, population, and age group. This review suggests that FA in HFM patients remain constant with time (age). All included studies were completed in countries such as the United States of America, China, Sweden, Italy, Finland and the Netherlands. Kearns et al. [24] and Kaprio et al. [39] used dentition age groups, Polley et al. [41] and Zhang et al. [43] utilised infancy to childhood age grouping, while others utilised various age groups in the included studies. FA was evaluated using parameters such as chin point, occlusal plane/angle, gonial/intergonial angle, piriform angle, ramus height, mandibular length, and body length [24, 25, 35–39]. The most reported parameter is ramus height, while the least reported parameter is piriform rim angle [24, 25, 35–39].

The mandible is the most affected facial bone in HFM, with FA reported as either increasing or remaining constant with age in HFM patients [25, 39, 42, 43]. Authors reporting progressive FA advocate for early surgical intervention to minimise end-stage deformity and psychosocial-economic impact, whereas others view FA as non-progressive, recommending surgical intervention at the end of growth due to the need for revisional surgery and increased psychosocial-economic burden [23, 25, 42]. The severity of the deformed mandible can be determined using various classification methods, with the Pruzansky or Pruzansky-Kaban classification being the gold standard for hypoplastic mandible in HFM [8, 44, 45]. Other classifications, such as SAT, OMENS, and OMENS+, were not used in the included studies [6, 9, 46]. Understanding these classifications ensures a proper treatment approach to mandibular lengthening through osteotomy, distraction osteogenesis, and grafting in maxillofacial or plastic surgery and orthodontics [47]. In addition to skeletal correction, structural fat grafts are used to augment soft tissue deformity in HFM patients, although this technique often requires multiple revisions [48]. New visualising techniques, including three-dimensional printed operation templates, offer advanced intervention planning despite challenges in treatment timelines and availability [49]. There are opposing views regarding the growth potential of the mandible in HFM patients. Some clinicians report the affected mandible becomes retarded in growth compared to the unaffected side [50], while others state the affected mandible grows parallel to the unaffected side [51, 52]. Evidence from dentition age groupings suggests an increase in deformity severity with age [24], although some studies, such as Polley and colleagues, indicate that the asymmetric mandible remains constant throughout growth [41]. Overall, while the growth pattern for different HFM severities appears non-progressive, severe HFM cases show otherwise [43]. This is crucial for diagnosing and managing HFM patients [24, 41, 53]. Also, it is noteworthy that rotations can still increase in one axis and decrease in another even when there are no overall growth changes [54]. An inherent genetic factor may cause asymmetry and increased angular measurement in FA [42]. However, some studies report that chin point values remain constant with age [25]. It is recommended that mandibular asymmetry correction in HFM be carried out during the mixed dentition age (6–12 years), though this is not the primary focus of this review [4, 55].

Assessment of HFM can be done either quantitatively or qualitatively [4] to determine the progressiveness of FA. Researchers have used parameters such as chin point, occlusal plane/angle, gonial/intergonial angle, piriform rim angle, ramus height, mandibular body length and total mandibular length to ascertain the extent of FA in HFM patients. Individual assessment of the indicators of FA on either progressive or non-progressive from the included articles in this review favoured the more non-progressive nature of HFM compared to few studies that favoured the progressive nature of the condition. Some authors have hypothesised that early treatment of HFM could promote midfacial growth and facial symmetry [3, 56]. At the same time, a report by Pluijmers et al. [57] does not support the hypothesis of the long-term stability of early treatment in HFM patients.

All the included studies in this review were completed in countries such as the United States of America, China, and various European nations; none were from Africa. Although there is a large body of existing literature on HFM, reports are primarily from international populations, with a few from the African continent [58–62]. It does not translate to Africa being spared of the burden of this congenital anomaly.

Psychological and biological features influence sexual dimorphism. Facial sexual dimorphism becomes more distinct after puberty due to the increased levels of androgen and oestrogen [63]. The shape difference decreases with age as the female mandible becomes like the male mandible while both sexes gain size [64]. Evidence from the included study shows no relationship between FA and gender dimorphism in the HFM population. Correspondingly, Nagy et al. [65] reported a similar distribution between sexes and dismissed the concept of prevalence in male patients and laterality to the right side. There is no sexual dimorphism in both progressive and non-progressive FA from the included studies. Therefore, there is no difference in treatment approach to HFM in both sexes. FA is typically found in humans; this becomes obvious in HFM patients due to the apparent defect to the mandible and associated facial skeleton [36, 42]. Multi-interventional approaches by healthcare providers such as otolaryngologists, audiologists, orthodontics, orthognathic surgeons, plastic surgeons, medical geneticists, and clinical psychologists are needed to manage and treat HFM.

The limitations of this review include the use of heterogenous age grouping, small sample size, less reporting on sex distribution in the sample size, insufficient empirical data on the severe skeletal or soft tissue deviation, the use of orthopantomograms to measure mandibular growth in some of the included studies may not cover the true depth of three-dimensional growth of the mandible and inconsistent use of angular measurement of FA in the included studies do not allow us to explore further facial asymmetry progression in other eligible studies. Furthermore, there is a lack of robust studies in the field of HFM; thus, no definitive conclusions can be drawn regarding the collective characteristics of this anomaly. All the included articles were longitudinal [25, 35, 38, 40, 42], except two [24, 43]. In the study reported by Shetye et al. [42], there is confusion regarding using the terms rate and ratio between the affected and unaffected sides in the HFM. Many of the eligible studies are case series studies [57]. Case series studies are regarded as low levels of evidence-based medicine. Therefore, caution is required to make firm conclusions when interpreting the information from these types of studies [57].

## Conclusion

The included studies widely use the Pruzansky or Pruzansky-Kaban classification systems. There is a correlation between progressive FA and dentition-age grouping. The FA parameters used in this review include chin point, gonial/intergonial angle, piriform angle, mandibular ramus height, mandibular length, and body length. An assessment of the included studies on FA parameters favoured more on the non-progressive nature of HFM. American and European populations have reported more on the progression of FA, with fewer reports from the Asian population. Evidence from the included studies indicates that FA does not increase in HFM patients, as demonstrated by the constant ratio of the affected side compared to the non-affected side during growth. The substantial establishment of a significant increase in FA with age in HFM remains uncertain. Hence, we premise that there is no progression of FA in HFM patients. However, the timing of the surgical intervention should be based on the functional or aesthetic needs of the patients. HFM patients with functional requirements such as obstructive sleep apnoea and feeding difficulty due to micrognathia or retrognathia may require interventions before skeletal maturity. The decision to operate on HFM prior to or after skeletal maturity should be individual-specific, considering both functional and psychosocial factors. More data are required from African and Asian populations for scholarly contribution to managing and treating HFM.

## Electronic supplementary material

Below is the link to the electronic supplementary material.

Supplementary Material 1

## Data Availability

No datasets were generated or analysed during the current study.

## Appendix I: search strategy

### MEDLINE via PubMed on 15th July 2022

PubMed 15/07/2022 = 625.

(((((((((“hemifacial microsomia” AND “facial asymmetry”) OR (“hemifacial microsomia” AND “mandibular growth restriction”)) OR (“hemifacial microsomia” AND “mandibular growth”)) OR (“hemifacial microsomia” AND “mandibular hypoplasia”)) OR (“hemifacial microsomia” AND “mandibular asymmetry”)) OR (“hemifacial microsomia” AND “mandibular morphology”)) OR (“facial asymmetry” AND “mandibular morphology”)) OR (“facial asymmetry” AND “mandibular hypoplasia”)) OR (hemifacial microsomia AND progressive facial asymmetry)) OR (progressive facial asymmetry AND mandibular asymmetry) NOT (review[pt])))))))))

## Abbreviations

FA: Facial Asymmetry
HFM: Hemifacial Microsomia
MMAT: Mixed Method Appraisal Tool
OMENS (+): Orbit distortion, Mandible hypoplasia, Ear anomaly, Nerve involvement, Soft tissue deficiency, Extracraniofacial defects
PRISMA: Preferred Reporting Items for Systematic reviews and Meta-analyses
SAT: Skeletal Auricular Soft tissue
USA: United States of America
WHO: World Health Organization

## Declarations

### Additional information

Correspondence and requests for materials should be addressed to LL.
